# Shape–Texture Debiased Training for Robust Template Matching †

**DOI:** 10.3390/s22176658

**Published:** 2022-09-02

**Authors:** Bo Gao, Michael W. Spratling

**Affiliations:** Department of Informatics, King’s College London, London WC2R 2LS, UK

**Keywords:** template matching, convolutional neural networks, VGG19

## Abstract

Finding a template in a search image is an important task underlying many computer vision applications. This is typically solved by calculating a similarity map using features extracted from the separate images. Recent approaches perform template matching in a deep feature space, produced by a convolutional neural network (CNN), which is found to provide more tolerance to changes in appearance. Inspired by these findings, in this article we investigate whether enhancing the CNN’s encoding of shape information can produce more distinguishable features that improve the performance of template matching. By comparing features from the same CNN trained using different shape–texture training methods, we determined a feature space which improves the performance of most template matching algorithms. When combining the proposed method with the Divisive Input Modulation (DIM) template matching algorithm, its performance is greatly improved, and the resulting method produces state-of-the-art results on a standard benchmark. To confirm these results, we create a new benchmark and show that the proposed method outperforms existing techniques on this new dataset.

## 1. Introduction

Template matching is a technique for finding a rectangular region of an image that contains a certain object or image feature. It is widely used in many computer vision applications, including object tracking [1,2], object detection [3,4], and 3D reconstruction [5,6]. A similarity map is generally used to quantify how well a template matches each location in an image, typically generated by sliding the template through the search image, then the matching position is determined by finding the location of maximum value of the similarity map. Traditional template matching generates the similarity map based on pixel intensity values, and is not robust to hard matching scenarios such as significant non-rigid deformations of the object, changes in the illumination and size of the target, and occlusion [7]. To address this issue, more distinctive hand-crafted features such as scale-invariant feature transform (SIFT) [8] and histogram of oriented gradients (HOG) [9] can be used instead of the intensity values for robust template matching [10,11,12,13]. However, these features must be extracted by certain manually predefined algorithms based on expert knowledge, and therefore have limited description capabilities [14].

With the help of deep features learned from convolutional neural networks (CNNs), vision tasks such as image classification [15,16], object recognition [17,18], and object tracking [19,20] have recently achieved great success. In order to succeed in such tasks, CNNs need to be trained with big data and automatically build internal representations that are less effected by changes in the appearance of objects in different images. Therefore, CNNs have strong description capability far exceeding that of hand-crafted features; recent methods have been successfully applied to a feature space produced by the convolutional layers of a CNN, achieving impressive performance [7,21,22,23,24,25,26].

The higher layers of CNNs are believed to learn representations of shapes from low-level features [27]. However, recent studies [28,29] have demonstrated that ImageNet-trained CNNs are biased toward making categorisation decisions based on texture rather than shape. The same works showed that CNNs could be trained to increase sensitivity to shape, resulting in improved accuracy and robustness of object classification and detection. Assuming that shape information is useful for template matching, these results suggest that the performance of template-matching methods applied to CNN-generated feature spaces could potentially be improved by training the CNN to be more sensitive to shape.

In this article, we verified this assumption by comparing features from five CNN models that had the same network structure while differing in shape sensitivity. Our results show that training a CNN to learn about texture while biasing it to be more sensitive to shape information can improve template matching performance. Furthermore, by comparing template-matching performance when using feature spaces created from all possible combinations of one, two, and three convolutional layers of the CNN, we found that the best results were produced by combining features from both early and late layers. Early layers of a CNN encode lower-level information such as texture, while later layers encode more abstract information such as object identity. Hence, both sets of results (the need to train the CNN to be more sensitive to shape and the need to combine information for early and late layers) suggest that a combination of texture and shape information is beneficial for template matching.

Our main contributions are summarised as follows:

• We created a new benchmark; compared to the existing standard benchmark, it is more challenging, provides a far larger number of image pairs, and is better able to discriminate between the performance of different template matching methods.
• By training a CNN to be more sensitive to shape information and combining features from both early and late layers, we created a feature space in which the performance of most template matching algorithms is improved.
• Using this feature space together with an existing template matching method, DIM [30], we obtained state-of-art results on both the standard and new datasets.

This paper is an extension of work originally presented at ICIVS2021 [31]. The conference paper reported the template matching results of the DIM algorithm using features extracted from four VGG19 models with different shape sensitivities in order to determine the best deep feature space for template matching, then compared the performance of many template-matching algorithms in that feature space. The current work adds a reviews of the latest literature in Section 2, details of the DIM algorithm in Section 3.2, new results using features from a new VGG19 model (Model_E) trained by the latest shape–texture debiased training method [29] along with related discussion in Section 4, visualisation of the results of different template matching algorithms in Section 4.4, and a concluding discussion in Section 5.

## 2. Related Work

### 2.1. Template Matching

Traditional template matching methods calculate the similarity map using a range of metrics such as the normalised cross-correlation (NCC), sum of squared differences (SSD), and zero-mean normalised cross-correlation (ZNCC), which are applied to the pixel intensity or colour values. However, because these methods rely on comparing the values in the template with those at corresponding locations in the image patch, they are sensitive to changes in lighting conditions, non-rigid deformations of the target object, or partial occlusions, which can result in a low similarity score when one or multiple of these situations occur. To overcome the limitations of classic template matching methods, many approaches [7,21,22,24,25,26,32] have been developed. These methods can be classified into two main categories.

One category attempts to increase tolerance to changes in appearance by changing the computation that is performed to compare the template to the image. For example, Best-Buddies Similarity (BBS) counts the proportion of sub-regions in the template and the image patch that are Nearest-Neighbour (NN) matches [7]. Deformable Diversity Similarity (DDIS) explicitly considers possible template deformation using the diversity of NN feature matches between a template and a potential matching region in the search image [24]. Annulus Projection Transformation and Neighbour Similarity (APT-MNS) [26] builds the global spatial structure of the target object using a novel annulus projection transformation (APT) vector to filter out the incorrectly matched NN candidates, then estimating the best matched candidates using the MNS measurement. Weighted Smallest Deformation Similarity (WSDS) [25] calculates the smallest deformation between each point in the template and its NN matches to explicitly penalise the deformation. In addition, weights are defined for points in the template based on their likelihood of belonging to the background calculated through NN matching with the points around the target window. This reduces the negative effect of background pixels contained in the template box. The Divisive Input Modulation (DIM) algorithm [30] extracts additional templates from the background and lets the templates compete with each other to match the image. Specifically, this competition is implemented as a form of probabilistic inference known as explaining away [33,34], which causes each image element to only provide support for the template that is the most likely match. Previous work has demonstrated that DIM, when applied to colour feature-space, is more accurate in identifying features in an image compared to both traditional and recent state-of-the-art matching methods [30].

The second category of approaches changes the feature space in which the comparison between the template and the image is performed. The aim is for this new feature space to allow better discrimination in template matching while increasing tolerance to changes in appearance. Co-occurrence based Template Matching (CoTM) transforms the points in the image and template to a new feature space defined by the co-occurrence statistics to quantify the dissimilarity between a template and an image [22]. Quality-Aware Template Matching (QATM) is a method that uses a pretrained CNN model as a feature extractor. It learns a similarity score that reflects the (softness) repeatness of a pattern using an algorithmic CNN layer [21]. Occlusion Aware Template Matching (OATM) [32] searches neighbours among two sets of vectors and uses a hashing scheme based on consensus set maximisation, and is hence able to efficiently handle high levels of deformation and occlusion.

### 2.2. Deep Features

Many template matching algorithms from the first category above can be applied both to deep features and directly to colour images. The deep features used by BBS, CoTM, and QATM are extracted from two specific layers of a pre-trained VGG19 CNN [35], *conv1_2* and *conv3_4*. Following the suggestion in [20] for object tracking, DDIS takes features from a deeper layer, fusing features from layers *conv1_2*, *conv3_4*, and *conv4_4*. In [23], the authors proposed a scale-adaptive strategy to select a particular individual layer of a VGG19 to use as the feature space according to the size of template. In each case, using deep features was found to significantly improve template matching performance compared to using colour features.

A recent study has shown that ImageNet-trained CNNs are strongly biased towards recognising textures rather than shapes [28]. The same study demonstrated that the same standard architecture (ResNet-50) [36] that learns a texture-based representation on ImageNet is able to learn a shape-based representation when trained on ‘Stylised-ImageNet’, a version of ImageNet that replaces the texture in the original image with the style of a randomly selected painting through AdaIN style transfer [37]. This new shape-sensitive model was found to be more accurate and robust in both object classification and detection tasks. However, the stylised dataset needs to be generated before the training process using a pre-defined set of texture source images. Due to computation and resource limitations, each image in the stylised dataset is only transferred by one random artistic image, which results in lack of diversity for each sample. Furthermore, the training process is complicated and involves first training the network on both standard and stylised training datasets, then fine-tuning on the standard dataset. In contrast, [29] proposes a shape–texture debiased training method which provides the corresponding supervisions from shape and texture simultaneously. Similarly, this method is based on AdaIN style transfer, with the difference in implementation being that it replaces the original texture information with uninformative texture patterns from another randomly selected image from the training mini-batch rather than with the style of randomly selected artistic paintings. This results in increments of diversity for each image; hence, this method achieves higher accuracy and robustness than [28] for image classification with the ResNet-50 architecture. Inspired by these findings, in this paper we investigate whether enhancing the shape sensitivity of a CNN can produce more distinguishable features that improve the performance of template matching.

## 3. Methods

### 3.1. Training CNN with Stylised Data

Previous work on template matching in deep feature space (see Section 2) has employed a VGG19 CNN. To enable a fair comparison with those previous results, we used the VGG19 architecture as well. However, we used five VGG19 models that differed in terms of the way they were trained to encode different degrees of shape selectivity, as summarised in Table 1.

Model_A to Model_D were trained using the same approach as in [28], with a stylised dataset generated before the training process; the ranking shape-sensitivity of these models was controlled by setting the different training datasets manually. Model_A was trained using the standard ImageNet dataset [35]; we used the pretrained VGG19 model from the PyTorch torchvision library. This model has the least shape bias. Model_B was trained on the Stylised-ImageNet dataset, and thus has the most shape bias. Model_C was trained on a dataset containing the images from both ImageNet and Stylised-ImageNet. Model_D was initialised with the weights of Model_C followed by fine-tuning on ImageNet for 60 epochs using a learning rate of 0.001 multiplied by 0.1 after 30 epochs. Therefore, Model_C and Model_D have intermediate levels of shape bias, with model_D being less selective to shape than Model_C. The stylised data samples used in Model_E were generated during training, and the training process provided supervisions from shape and texture simultaneously [29]. Hence, Model_E has an intermediate level of shape bias, although where it should rank relative to Model_C and Model_D was impossible to quantify. The learning rate was 0.01 multiplied by 0.1 after every 30 epochs for Model_B and Model_E, and after every 15 epochs for Model_C. The number of epochs was 90 for Model_B and Model_E and 45 for Model_C; as the dataset used to train Model_C was twice as large as that used to train Model_B, the number of weight updates was the same for both models. The other training hyperparameters used for each model were a batch size of 256, momentum 0.9, and weight decay 1 × 10^−4^. The optimiser was SGD.

### 3.2. DIM Template Matching Algorithm

The DIM algorithm has previously been found to produce the best performance for template matching in colour feature space [30]. Hence, it was selected as the underlying algorithm to determine the best CNN feature space to use for template matching. A detailed description of the DIM algorithm can be found in [30]; for the convenience of the reader, a brief introduction is provided below.

**Figure 1:**
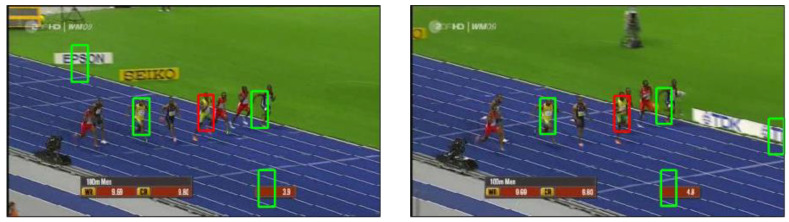
An illustration of template competition. The red rectangle area in the left image shows the template used for finding the matching location in the right image. The four same-sized green rectangle areas are the additional templates taken from the left image. These templates compete with each other to be matched to the right image. This competition means that only one template can be the best matching one at each location. The locations matching each template are shown on the right.

In contrast to other template matching methods that only use the appearance of the target, DIM considers potential distractors, that is, regions that are similar to the matching target. These distractors are represented as additional templates that are cropped from the same image as the given template. All of the templates, representing both the target and the distractors, compete with each other to be matched with the search image. This inference process performs by explaining away [33,38,39]: possible causes (i.e., templates) compete to explain the sensory evidence (i.e., the search image), and if one cause explains part of the evidence (i.e., a part of the image), then support from this evidence for alternative explanations (i.e., other templates) is reduced, or explained away. An example is shown in Figure 1.

DIM minimises the Kullback–Leibler (KL) divergence between the input and a reconstruction of the input created by the additive combination of the templates. This requires the input to be non-negative [30]. Therefore, a pre-processing step is required to separate the positive and rectified negative values of the features directly into two parts, which are then concatenated along the channel dimension:

(1) Ipre=concatenate(ReLU(ϕ(I)),ReLU(−ϕ(I)))

where I is a colour or grayscale image, ϕ(I) are features extracted from the image, and ReLU is the function that, if positive, outputs an element of the input directly, and otherwise outputs zero.

To apply DIM directly to the image feature space, feature extraction was performed as follows:

(2) $$\phi \left(I\right)=\gamma \sum_{c=1}^{k}\left(I_{c}-I_{c}🟉f\right)$$

where *c* is the index over the number of image channels, *k* has a value of one for a grayscale image and three for a colour image, γ is a gain factor that was set here to a value of 2, and *f* is a Gaussian filter with a standard deviation equal to half of the smaller value of the template width or height [30]. This operation results in each channel of ϕ(I) being represented by the deviations between the pixel intensity values and the local mean intensity. In this paper, the five VGG19 models were used as feature extractors; hence, ϕ(I) represents deep features of I extracted by the CNNs.

Both the template and the search image were pre-processed as described in the previous paragraph. For the template image, additional templates were extracted around locations where the correlation between the target template and the image was strongest, excluding locations where the additional templates would overlap with each other or where the bounding box defined the target [30]. Five additional templates were used in the experiments described in this paper. DIM requires the templates to have dimensions that are odd numbers, otherwise the reconstruction of the input does not align with the actual input; see Equation (3) for details. Therefore, if one side of the target template is even, it is padded by one row on the right or one column on the bottom with zeros, then the new size of target template is used to generate the additional templates.

DIM was implemented using the following equations:

(3) $$R_{i}=\sum_{j=1}^{p}\left(v_{ji}🟉S_{j}\right)$$

(4) $$E_{i}=Ipre_{i}⊘{\left[R_{i}\right]}_{\epsilon_{2}}$$

(5) $$S_{j}\leftarrow {\left[S_{j}\right]}_{\epsilon_{1}}⊙\sum_{i=1}^{k}\left(w_{ji}*E_{i}\right)$$

where *i* is an index over the number of input channels (the maximum index *k* is twice the channel number of the extracted features); *j* is an index over *p*, which is the number of different templates being compared to the image; $R_{i}$ is a two-dimensional array representing a reconstruction of $Ipre_{i}$ (I, pre-processed using Equation (1)); $E_{i}$ is a two-dimensional array representing the discrepancy (or residual error) between $Ipre_{i}$ and $R_{i}$; $S_{j}$ is a two-dimensional array that represents the similarity between template feature *j* and the image feature at each pixel; $w_{ji}$ is a two-dimensional array representing channel *i* of template *j*, with the sum of the values in each template $w_{j}$ being normalised in order to sum to one; $v_{ji}$ is another two-dimensional array representing template values (where the values of $v_{j}$ were made equal to the corresponding values of $w_{j}$, except that they were normalised to have a maximum value of one); ${\left[\cdot \right]}_{\epsilon }=max\left(\cdot ,\epsilon \right)$; $\epsilon_{1}$ and $\epsilon_{2}$ are parameters with their values set to $\frac{\epsilon_{2}}{\max(\sum_{j}^{p}v_{ji})}$ and $1\times 10^{-2}$, respectively; ⊘ and ⊙ indicate element-wise division and multiplication, respectively; and 🟉 and * represent the cross-correlation and convolution operations, respectively. All elements of S were initially set to zero, and Equations (3)–(5) were iteratively updated and terminated after ten iterations for all of the experiments reported in this paper.

For a search image I, in order to avoid a poor estimate of ϕ(I) and edge effects during template matching, when DIM was directly applied to the image feature-space, I was first padded on all sides with intensity values that were mirror reflections of the image pixel values near the borders of I. The width of the padding was equal to the width of the template on the left and right borders and equal to the height of the template on the top and bottom borders. The final similarity maps S were cropped to be the same size as the original image once the template matching method had been applied [30]. When applying DIM to deep feature space, ϕ(I) was padded using the same method corresponding to the width and height of the template in deep space, and S was cropped to be the same size as ϕ(I) after application of DIM.

The best matching location can be represented by a single element with the largest value of the similarity map $S_{j}$ for template *j*, as in other template matching methods. However, the best matching location is often represented by a small population of neighbouring elements with high values [30]. Therefore, post-processing was performed to sum the similarity values within neighbourhoods:

(6) $$S_{j}=S_{j}🟉K_{e}\left(max(1,\alpha *w),max(1,\alpha *h)\right)$$

where $K_{e}$ is a binary-valued kernel containing ones within an elliptically shaped region, and has a width and height equal to α times the width *w* and height *h* of the template; α was set to 0.025.

## 4. Results

### 4.1. Dataset Preparation

The BBS dataset [7] has been widely used for the quantitative evaluation of template matching algorithms [7,21,22,23,24]. This dataset contains 105 template–image pairs sampled from 35 videos (three pairs per video) from a tracking dataset [40]. Each template–image pair is taken from frames of the video that are 20 frames apart. To evaluate the performance of a template matching algorithm, the intersection-over-union (IoU) is calculated between the predicted bounding box and the ground truth box for the second image in the pair. The overall accuracy is then determined by calculating the area under the curve (AUC) of a success curve produced by varying the threshold of IoU that counts as success.

**Figure 2:**
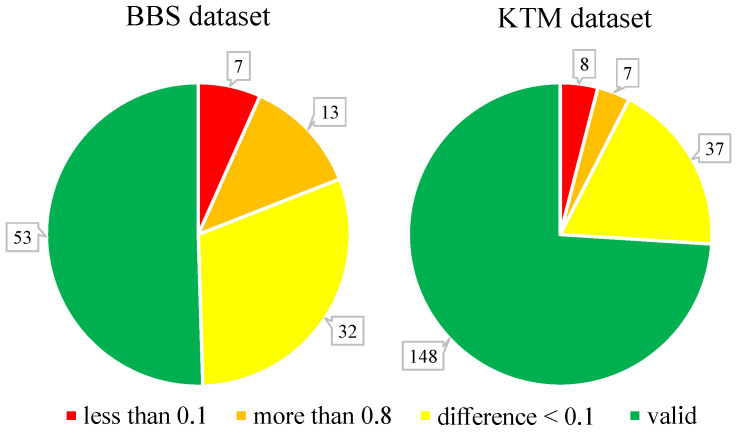
Discriminative ability of two datasets evaluated by comparing the IoU scores produced by ZNCC, BBS, DDIS, and DIM.

Although the BBS data is widely used, it is not particularly good at discriminating the performance of different template matching methods. To illustrate this issue, we applied one baseline method (ZNCC) and three state-of-art methods (BBS, DDIS, and DIM) to the BBS dataset in colour space. The results show that there are 52 template–image pairs where all methods generate very similar results; these can be sub-divided into seven template–image pairs for which all methods fail to match (IoU less than 0.1 for all four methods), 13 template–image pairs for which all methods succeed (IoU greater than 0.8 for all four methods), and 32 template–image pairs for which all methods produce similar intermediate IoU values within 0.1 of each other. This means that only 53 template–image pairs in the BBS dataset help to discriminate the performance of these four template matching methods. These results are summarised in Figure 2.

We therefore created a new dataset, the King’s Template Matching (KTM) dataset, following a similar procedure to that used to generate the BBS dataset. The new dataset contains 200 template–image pairs sampled from 40 new videos (five pairs per video) selected from a different tracking dataset [41]. In contrast to the BBS dataset, the template and the image were chosen manually in order to avoid pairs that contain significant occlusions and non-rigid deformations of the target (which no method is likely to match successfully), and the image pairs were separated by 30 (rather than 20) frames in order to reduce the number of pairs for which matching would be easy for all methods. These changes make the new data more challenging and provide a far larger number of image pairs that can discriminate the performance of different methods, as shown in Figure 2. Both the new dataset and the BBS dataset were used in the following experiments.

### 4.2. Template Matching Using Features from Individual Convolutional Layers

**Figure 3:**
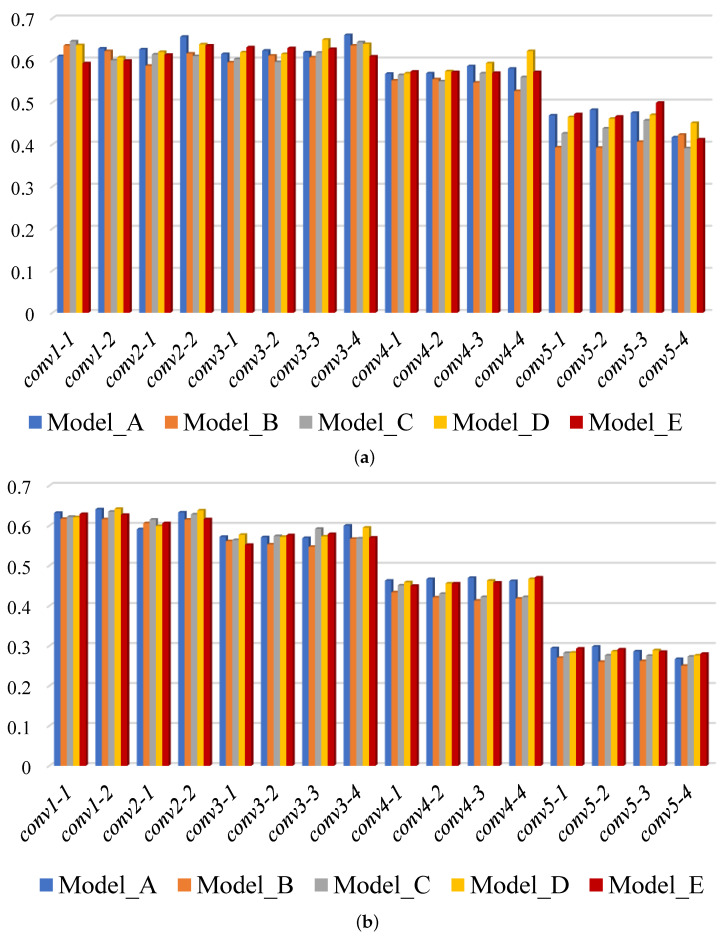
The AUC scores of DIM using features from different convolutional layers of five models: (**a**) evaluation on BBS dataset and (**b**) evaluation on KTM dataset.

**Figure 4:**
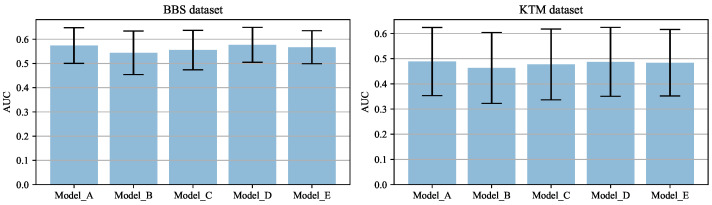
Mean and standard deviation of the AUC scores using features from different convolutional layers of five models.

To reveal how the shape bias affects template matching, we calculated the AUC using DIM with features from every single convolutional layer of the five models. As the features from the later convolutional layers are down-sampled using max-pooling (by a factor of $\frac{1}{2}$, $\frac{1}{4}$, $\frac{1}{8}$, and $\frac{1}{16}$ compared to the original image), the bounding box of the template was multiplied by the same scaling factor and the resulting similarity map is resized back to the original image size in order to make the prediction. The AUC scores across the BBS and KTM datasets are summarised in Figure 3, and the mean and standard deviation of these AUC scores are summarised in Figure 4.

For all five models, there is a tendency for the AUC to be higher when template matching is performed using lower layers of the CNN compared to later layers. This suggests that template matching relies more on low-level visual attributes such as texture, rather than higher-level ones such as shape. Among the four models trained with stylised samples, the AUC score for most CNN layers is greater for Model_D than Model_E, greater for Model_E than Model_C, and greater for Model_C than Model_B. This tendency, which can be clearly seen in Figure 4, suggests that template matching relies more on texture features than shape features. Comparing Model_A and Model_D, it is hard to say which is better. However, the AUC score calculated on the BBS dataset using features from *conv4_4* of Model_D is noticeably better than that for Model_A. This suggests that increasing the shape bias of later layers of the CNN could potentially lead to better template matching. However, this result is not reflected by the results for the KTM dataset. One possible explanation is that, in general, the templates in the KTM dataset are smaller than those in the BBS dataset (with the template size defined in terms of area, i.e., as the product of its width and height; the mean template size of for the KTM dataset is 1603 pixels${}^{2}$, whereas it is 3442 pixels${}^{2}$ for the BBS dataset). Smaller templates tend to be less discriminative. The sub-sampling that occurs in later levels of the CNN results in templates that are even smaller and less disriminative. This may account for the worse performance of the later layers of each CNN when tested using the KTM dataset rather than the BBS dataset. This represents a a confounding factor in attributing the better performance of the early layers to a reliance on texture information.

**Figure 5:**
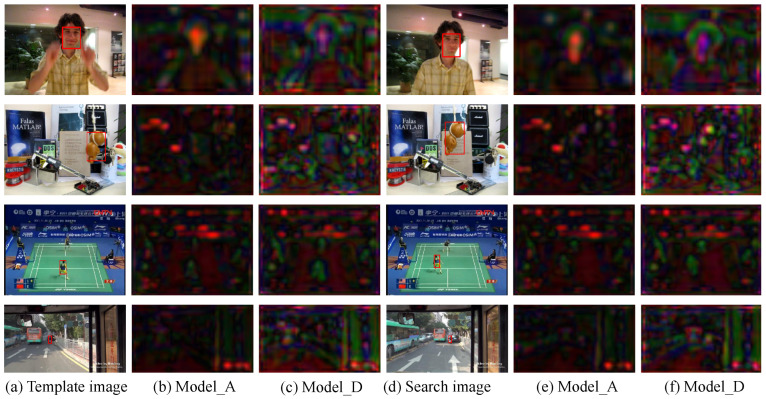
Visualisation of *conv4_4* layers for image pairs with different template sizes: (**a**) the first image in the pair shows the bounding box defining the template; (**b**,**c**) visualisations of the corresponding features produced in layer *conv4_4* of (**b**) Model_A and (**c**) Model_D; (**d**) the second image in the pair shows the bounding box of the ground truth location of the target object; (**e**,**f**) visualisations of the corresponding features produced in layer *conv4_4* of Model_A and Model_D, respectively.

In order to illustrate the differences in the features learned by Model_A and Model_D, the first three principal components of *conv4_4* were converted to RGB values. As shown in Figure 5, the features from Model_D contain more information about edges (shape) than those from Model_A. However, it is hard to distinguish the small object in fourth row, as it is represented by a very small region of the feature space.

### 4.3. Template Matching Using Features from Multiple Convolutional Layers

We compared Model_A, Model_D, and Model_E by applying the DIM template matching algorithm to features extracted from multiple convolutional layers of each CNN. In order to combine feature maps with different sizes, bilinear interpolation was used to make them the same size. If the template was small (height times width less than 4000) the feature maps from the later layer(s) were scaled to be the same size as those in the earlier layer(s). If the template was large, the feature maps from the earlier layer(s) were reduced in size to be the same size as those in the later layer(s). To maintain a balance between low and high level features, the dimension of the feature maps from the later layer(s) was reduced by PCA to the same number as in the earlier layer.

Table 2 shows the AUC scores produced by DIM using features from two convolutional layers of Model_A, Model_D, and Model_E. All possible combinations of two layers were tested; the table shows only selected results with the best performance. It can be seen from Table 2 that for the 24-layer combinations for which results are shown, 21 results for both BBS and KTM dataset are better for Model_D than for Model_A, and 14 results for BBS dataset and 13 results for KTM dataset are better for Model_E for Model_A. Hence, both networks with more shape bias perform better than the network with the least shape bias. These results thus support the conclusion that more discriminative features can be obtained by increasing the shape bias of the VGG19 model, which increases the performance of template matching.

The results for Model_D are better than those for Model_E for 17 of the 24 layer combinations for the BBS dataset and for 18 of the 24 layer combinations for the KTM dataset. Furthermore, the best result for each dataset (indicated in bold) is generated using the features from Model_D. Hence, among the three models, Model_D produced best performance. To determine whether fusing features from more layers would further improve template matching performance, DIM was applied to all combinations of three layers from Model_D, resulting in a total of 560 different combinations using three layers. As it is impossible to show all these results in this paper, the highest ten AUC scores are shown in Table 3. For both datasets, using three layers produced an improvement in the best AUC score (around 0.01) compared to using two layers.

### 4.4. Comparison with Other Methods

This section compares our results with those produced by other template matching methods in both colour and deep feature space. When evaluated on the BBS dataset, the deep features used by each template matching algorithm were the features from layers *conv1_2*, *conv4_1*, and *conv4_4* of Model_D. When evaluated on the KTM dataset, the deep features used as the input to each algorithm were those from layers *conv1_1*, *conv3_4*, and *conv4_2* of Model_D. BBS, CoTM, and QATM have been tested on BBS data by their authors using different deep features, and thus we compared our results to these earlier published results as well.

**Figure 6:**
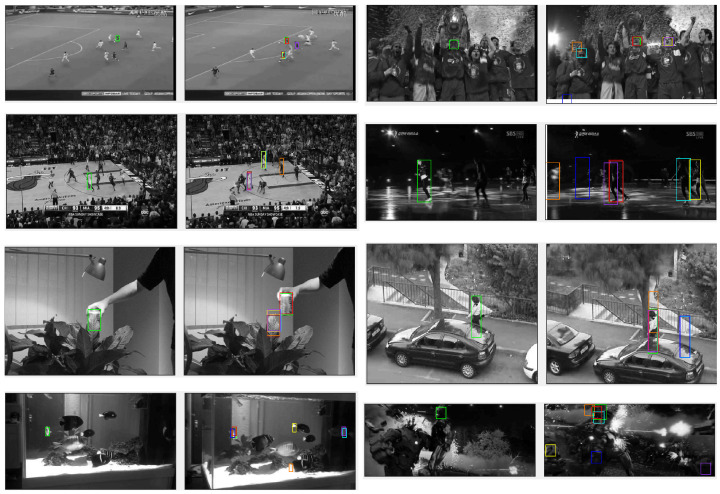
Comparison performance of different methods using the proposed feature extraction method. The colour boxes indicate the template location defined by: ⎯⎯ Ground truth ⎯⎯ BBS ⎯⎯ CoTM ⎯⎯ DDIS ⎯⎯ DIM ⎯⎯ NCC ⎯⎯ QATM ⎯⎯ SSD ⎯⎯ ZNCC. Note that the colour images have been converted to greyscale in order to make the bounding boxes more visible. The boxes predicted by ZNCC and NCC are remarkably similar, and thus overlap.

The comparison results are summarised in Table 4, and examples of the results for particular images are shown in Figure 6. All methods except QATM and BBS produce improved results using the proposed deep features than when using colour features. This is true for both datasets. Of the methods that have previously been applied to deep features, the performance of two (NCC and QATM) are improved, while that of two others (BBS and CoTM) is made worse when using our proposed method to define the deep feature space. Potential further improvements to the performance of these methods could be achieved by optimising the feature extraction method for the individual template matching algorithm, as has been done here for DIM. However, it should be noted that simple metrics for comparing image patches such as NCC and ZNCC produce close to state-of-the-art performance when applied to our proposed deep feature space, outperforming much more complex methods of template matching such as BBS, CoTM, and QATM when these methods are applied to any of the tested feature spaces, including those proposed by the authors of these algorithms.

ZNCC and NCC produce very similar scores on both datasets. ZNCC is similar to NCC, with the only difference being the subtraction of the local mean value from the feature vectors being compared. This operation makes ZNCC more robust to changes of lighting conditions when applied directly to colour images, and for this reason the AUC score of ZNCC on both datasets is higher than that of NCC in colour space. The features extracted by the CNN appear to be insensitive to lighting changes; therefore, the results of NCC and ZNCC are remarkably similar when applied to these features.

One known weakness of BBS is that it may fail when the template is very small compared to target image [7]. This may explain the particularly poor results of this method when applied to the KTM dataset.

DIM achieves the best results on both datasets when applied to deep features. DIM performs particularly well on the BBS dataset, producing an AUC of 0.73, which, as far as we are aware, makes it the only method to have scored more than 0.7 on this dataset. The DIM algorithm produces state-of-the-art performance on the KTM dataset when applied to deep features. When applied to colour features, the results are good, although not as good as DDIS on the KTM dataset. This is because small templates in the KTM dataset may contain insufficient detail for the DIM algorithm to successfully distinguish the target object. Using deep features enhances the discriminatory ability of small templates enough that the performance of DIM increases significantly. These results demonstrate that the proposed approach is effective at extracting distinguishable features, which lead to robust and accurate template matching.

## 5. Discussion

The experiments described above demonstrate that template matching relies more on low-level visual attributes such as texture than higher-level attributes such as shape. However, it is clear that slightly increasing the shape bias of a CNN by changing the method of training the network and then combining the outputs of a range of convolutional layers produces a feature space in which template matching can be achieved with greater accuracy. This is because the combination of low-level features that can accurately locate the target with high-level features that are more tolerant to appearance changes enables more robust recognition and localisation of the target object.

## 6. Conclusions

Our results demonstrate that slightly increasing the shape bias of a CNN by changing the method used to training the network can produce more distinguishable features, allowing template matching to be achieved with greater accuracy. By running a large number of experiments using shape-biased VGG19 architectures, we determined the best combination of convolutional features on which to perform template matching with the DIM algorithm. This same feature space was shown to improve the performance of most other template matching algorithms as well. When applied to our new feature space, the DIM algorithm was able to produce state-of-art results on two benchmark datasets.

## Figures and Tables

**Table 1:** The five different VGG19 CNN models used in this paper. IN and SIN are the abbreviations of ImageNet and Stylised-ImageNet, respectively.

Name	Training	Fine-Tuning	Rank of Shape Sensitivity
Model_A	IN	-	4
Model_B	SIN	-	1
Model_C	IN + SIN	-	2
Model_D	IN + SIN	IN	3
Model_E	IN	-	-

**Table 2:** Partial AUC scores of DIM using features from two convolutional layers of Model_A (upper value in each cell), Model_D (middle value in each cell), and Model_E (lower value in each cell). The up and down arrows indicate whether the AUC score of Model_D or Model_E is better or worse than that of Model_A.

**(a) Evaluation on BBS dataset.**								
		Layer	conv3_3	conv3_4	conv4_1	conv4_2	conv4_3	conv4_4
	AUC							
Layer								
*conv1_1*			0.710	0.705	0.713	0.697	0.698	0.711
			0.707↓	0.714↑	0.704↓	**0.718**↑	0.710↑	0.708↓
			0.692↓	0.673↓	0.700↓	0.711↑	0.697↓	0.694↓
*conv1_2*			0.686	0.686	0.674	0.655	0.680	0.683
			0.686	0.687↑	0.707↑	0.696↑	0.690↑	0.710↑
			0.658↓	0.659↓	0.675↑	0.695↑	0.683↑	0.677↓
*conv2_1*			0.658	0.670	0.664	0.653	0.662	0.667
			0.659↑	0.669↓	0.665↑	0.671↑	0.683↑	0.693↑
			0.686↑	0.663↓	0.666↑	0.690↑	0.687↑	0.687↑
*conv2_2*			0.659	0.661	0.653	0.641	0.659	0.663
			0.665↑	0.667↑	0.676↑	0.679↑	0.676↑	0.682↑
			0.682↑	0.653↓	0.688↑	0.684↑	0.672↑	0.691↑
**(b) Evaluation on KTM dataset.**								
		Layer	conv3_3	conv3_4	conv4_1	conv4_2	conv4_3	conv4_4
	AUC							
Layer								
*conv1_1*			0.687	0.684	0.677	0.668	0.670	0.678
			0.689↑	0.691↑	0.682↑	0.695↑	0.684↑	0.687↑
			0.672↓	0.676↓	0.664↓	0.672↑	0.675↑	0.678
*conv1_2*			0.687	0.689	0.682	0.685	0.675	0.682
			0.680↓	0.694↑	0.695↑	**0.697**↑	0.691↑	0.690↑
			0.668↑	0.672↓	0.670↓	0.669↓	0.669↓	0.673↓
*conv2_1*			0.634	0.633	0.647	0.645	0.655	0.639
			0.642↑	0.651↑	0.665↑	0.671↑	0.668↑	0.666↑
			0.658↑	0.673↑	0.643↓	0.648↑	0.665↑	0.660↑
*conv2_2*			0.642	0.651	0.664	0.661	0.669	0.669
			0.657↑	0.664↑	0.670↑	0.673↑	0.669	0.669
			0.657↑	0.665↑	0.640↑	0.651↓	0.665↓	0.670↑

**Table 3:** Best ten results when using combinations of features from three convolutional layers of Model_D; here, for instance, $C12_{44}^{41}$ means that features from *conv1_2*, *conv4_1*, and *conv4_4* were fused.

(a) Evaluation on BBS dataset.										
Layers	$C12_{44}^{41}$	$C11_{43}^{34}$	$C11_{44}^{42}$	$C12_{52}^{43}$	$C11_{43}^{22}$	$C11_{44}^{41}$	$C11_{43}^{41}$	$C11_{42}^{34}$	$C12_{44}^{43}$	$C11_{44}^{34}$
AUC	0.728	0.727	0.724	0.724	0.723	0.722	0.720	0.720	0.720	0.720
**(b) Evaluation on KTM dataset.**										
Layers	$C11_{42}^{34}$	$C12_{43}^{32}$	$C11_{43}^{34}$	$C12_{42}^{22}$	$C12_{42}^{31}$	$C12_{42}^{34}$	$C12_{43}^{34}$	$C12_{43}^{31}$	$C12_{43}^{33}$	$C11_{42}^{33}$
AUC	0.711	0.709	0.708	0.706	0.706	0.705	0.705	0.705	0.705	0.704

**Table 4:** Quantitative comparison of the performance of different template matching algorithms using different input features.

		Feature	BBS Dataset			KTM Dataset	
	AUC		Colour	Deep	Deep (Proposed)	Colour	Deep (Proposed)
Method							
SSD			0.46	-	0.54	0.42	0.54
NCC			0.48	0.63 [23]	0.67	0.42	0.67
ZNCC			0.54	-	0.67	0.48	0.67
BBS			0.55	0.60 [7]	0.54	0.44	0.55
CoTM			0.54 ^1^	0.67 [22]	0.64	0.51	0.56
DDIS			0.64	-	0.66	**0.63**	0.68
QATM			-	0.62 ^2^	0.66	-	0.64
DIM			**0.69**	-	**0.73**	0.60	**0.71**

${}^{1}$ We were unable to reproduce this result using the code provided by the authors of CoTM. Our different result is shown in the table. ${}^{2}$ The authors of QATM report an AUC score of 0.69 when this method is applied to the BBS dataset [21]. However, examining their source code, we note that this result is produced by setting the size of the predicted bounding box as equal in size to the width and height of the ground truth bounding box. Other methods are evaluated by setting the size of the predicted bounding box equal to the size of the template (i.e., without using knowledge of the ground truth that the algorithm is attempting to predict). We have re-tested QATM using the standard evaluation protocol and our result for the original version of QATM is 0.62. As QATM is designed to work specifically with a CNN, it was not applied directly to colour images.

## Data Availability

As part of this study we created a new benchmark, KTM, for template matching. This dataset, along with the code used in the proposed method, can be found at: https://github.com/iminfine/Deep-DIM (accessed on 1 June 2022).
